# 19q13.11 microdeletion: Clinical features overlapping ectrodactyly ectodermal dysplasia‐clefting syndrome phenotype

**DOI:** 10.1002/ccr3.1600

**Published:** 2018-05-28

**Authors:** Kikue Terada Abe, Isabela M. P. O. Rizzo, Ana L. V. Coelho, Nilo Sakai, Daniel R. Carvalho, Carlos E. Speck‐Martins

**Affiliations:** ^1^ Cytogenetic Laboratory Molecular Pathology SARAH Network of Rehabilitation Hospitals Brasília Brazil; ^2^ Department of Clinical Genetics SARAH Network of Rehabilitation Hospitals Brasília Brazil

**Keywords:** chromosome 19q13.11 deletion syndrome, developmental disabilities, ectodermal dysplasia, intellectual disability

## Abstract

We report a patient who was followed for a long time under an ectrodactyly ectodermal dysplasia‐clefting (EEC) syndrome and was subsequently diagnosed with a 19q13.11 microdeletion. After a review of the related literature, we suggest testing patients with EEC for 19q13.11 microdeletion and include *WTIP* and *UBA2* to a minimal overlapping region.

## 1 INTRODUCTION

19q13.11 microdeletion syndrome (MIM 613026) is a clinically recognizable condition that has been recently identified using microarrays for genome‐wide screening as a comparative genomic hybridization (CGH) array or a chromosome microarray. To date, 14 cases have been reported of deletions in the 19q13.11 region^1^, ^2^, ^3^, ^4^, ^5^, ^6^, ^7^, ^8^, ^9^ and additional 4 cases annotated in the Database of Chromosome Imbalance and Phenotype in Humans using Ensembl Resources — DECIPHER (patients with loss‐definitely pathogenic: 127, 317705, 339538 and 351864).^10^ The main clinical characteristics are intrauterine and postnatal growth retardation, microcephaly, developmental delay/intellectual disabilities, language delay, feeding difficulties, slender habitus, cutis aplasia over the posterior occiput, and genital malformation in males (hypospadias). The first patient with those related characteristics presented an approximately 11‐megabase (Mb) deletion on 19q12q13.1.^1^ Most of the reported deletions were between 1.37 and 8.16 Mb in size, with the smallest region of overlap spanning a 324‐kb region in which four zinc finger (*ZNF*) genes were located.^5^ However, some authors have noted the importance of other genes that are proximal to the proposed minimal overlapping region (MOR) as the cause of major features.^3^, ^4^, ^7^, ^9^ More clinical reports and research on the functions of these genes are expected to support their contribution to the phenotype.

We report a 9‐year‐old male patient whose phenotype was initially attributed to EEC syndrome. Using a chromosomal microarray, we detected an interstitial deletion overlapping the 19q13.11 region and compared our findings with the patients reported in the literature.

## 2 CLINICAL REPORT

**Figure 1:**
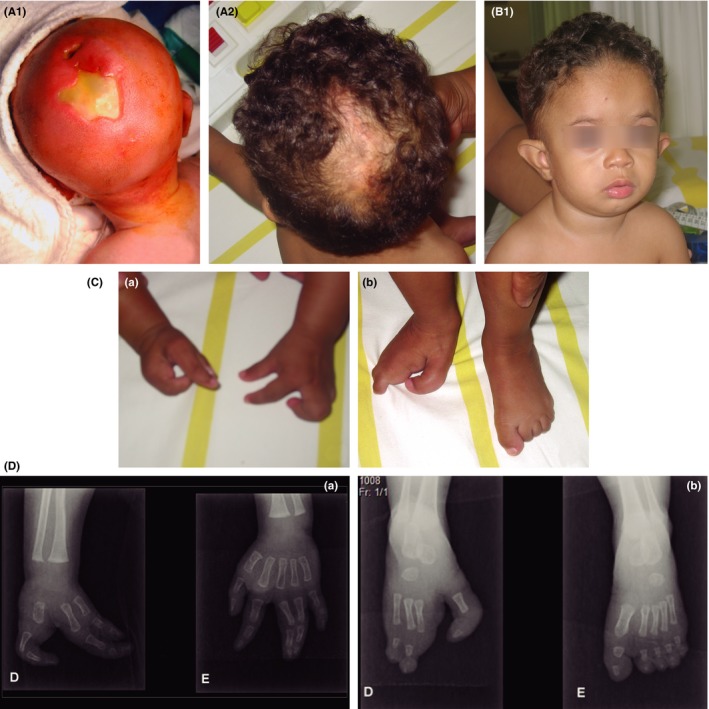
(A1) Patient at one day after birth, showing cutis aplasia in midline scalp before surgical correction; (A2) Cutis in midline scalp at one year and 20 days‐old after the correction of the cutis aplasia; (B1) Patient at one year and 20 days‐old, note high forehead, micrognathia, low set, and poorly folded ears; (C(a)) Note ectrodactyly on the hands; (C(b)) Ectrodactyly on the feet; (D(a)) Radiograph of right hand shows a typical cleft hand deformity with absence of the central rays (the third and fourth rays); radiograph of left hand shows a complete simple syndactyly of third and fourth rays. (D(b)) Radiograph of right foot shows a typical cleft feet deformity with complete absence of second ray, third metacarpal was present but phalanges of middle finger were absent; complete simple syndactyly of 4th and 5th rays, and lateral deviation of the hallux. Radiograph of left foot shows a partial simple syndactyly from second to fifth toes

The male child reported here is the second‐born child to nonconsanguineous, healthy parents (mother was 38 years old and father was 41 years old). He has one older healthy sister. He was born at 36 weeks of gestation by cesarean section, due to intrauterine growth retardation and oligohydramnios. His weight was 1790 g (<10th percentile), height was 45 cm (10th‐25th percentile) and occipitofrontal circumference (OFC) was 30 cm (<3rd percentile). The Apgar score was 7/8. At birth, parietal encephalocele, hypospadias, cutis aplasia in midline scalp, and ectrodactyly in hand and foot were noticed. There was median cleft in the right hand, syndactyly between the 1st and 2nd fingers and agenesis of the 3rd and 4th fingers; in the left hand, there was syndactyly between the 3rd and 4th fingers. The right foot presented median cleft, deformed of the hallux, and the partial absence of the 2nd and 3rd toes. He showed some minor facial dysmorphic features: high forehead, prominent ears, and retrognathia (Figure 1). The first diagnostic hypothesis was EEC syndrome because the rare association of congenital cutis aplasia and split hand/split foot malformations. Growth retardation, microcephaly, developmental delay, language delay, behavior disturbance, astigmatism, poor suck‐swallow coordination, gastroesophageal reflux, and upper airway allergy were observed in his early infancy. He underwent several surgical procedures to treat encephalocele, gallbladder stone, syndactyly, and hypospadias. In terms of image studies, the abdominal ultrasonography and echocardiogram provided normal results. Computerized tomography and magnetic resonance imaging of his brain yielded a posterior slit of sagittal suture and signs of Dandy‐Walker variation. Other tests as electroencephalogram (EEG), audiometry, thyroid‐stimulating hormone (TSH), thyroid hormone (T4) tests, and growth hormone also were normal. At 10 years old, the body measurements were below the percentile 1 (height 121 cm, weight 17.6 kg, and OFC 46 cm) associated with 1 year delay in bone age. It was noticed an irregular eruption in the upper and lower dental arch with normal enamel. Intellectual disability was diagnosed with the Wechsler Intelligence Scale for Children‐Fourth Edition (WISC‐IV). The treatment of penile hypospadias was in the course, and there was primary daytime incontinence.

**Figure 2:**
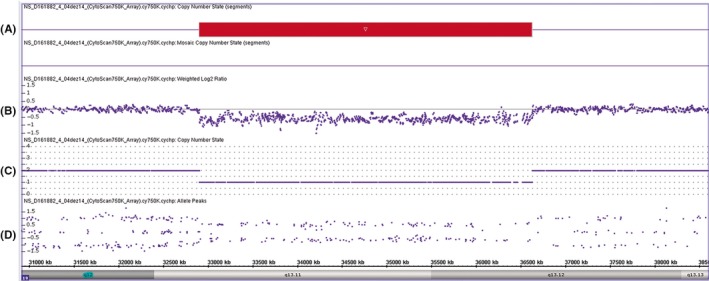
Array CGH profile of chromosome 19 showing deletion at 19q13.11: (A) Copy Number State segment (red box), (B) weighted log2 ratio, (C) copy number state, (D) allele peaks

We first performed conventional cytogenetic analysis to rule out complex chromosomal rearrangement, which showed a normal 46, XY karyotype, at age of one year and three months. At ten years old, he underwent to an oligo‐SNP array (Affymetrix CytoScan 750K array ‐ Santa Clara, CA, USA) performed on genomic DNA obtained from the peripheral blood sample. The resolution of this array is estimated at 1.15 kb. Thresholds for genome‐wide screening were set at >200 kb and >50/25 markers for gains/losses, respectively, and 10 kb for segments of homozygosity. The microarray data were processed and analyzed using Chromosome Analysis Suite (ChAS 2,0), version 33.1[r9069], provided by the manufacturer. A heterozygous 3.72‐Mb interstitial deletion at 19q13.11q13.12 (genomic position 32 904 200‐36 627 790) was identified (Human Genome Build 37/hg19, 2009) (Figure 2). The deleted region contains 95 genes (including non‐coding RNAs and hypothetical proteins) and 60 protein‐coding OMIM genes. This array result was interpreted as pathogenic, and no other remarkable genomic change was observed. Parental testing was not performed because their DNA was not available.

This study was approved by the National Committee of Ethics in Research, SARAH Network of Rehabilitation Hospitals. The parents of the patient signed informed consent and approved the anonymous use of clinical and molecular data for the present diagnostic study.

## 3 DISCUSSION

**Figure 3:**
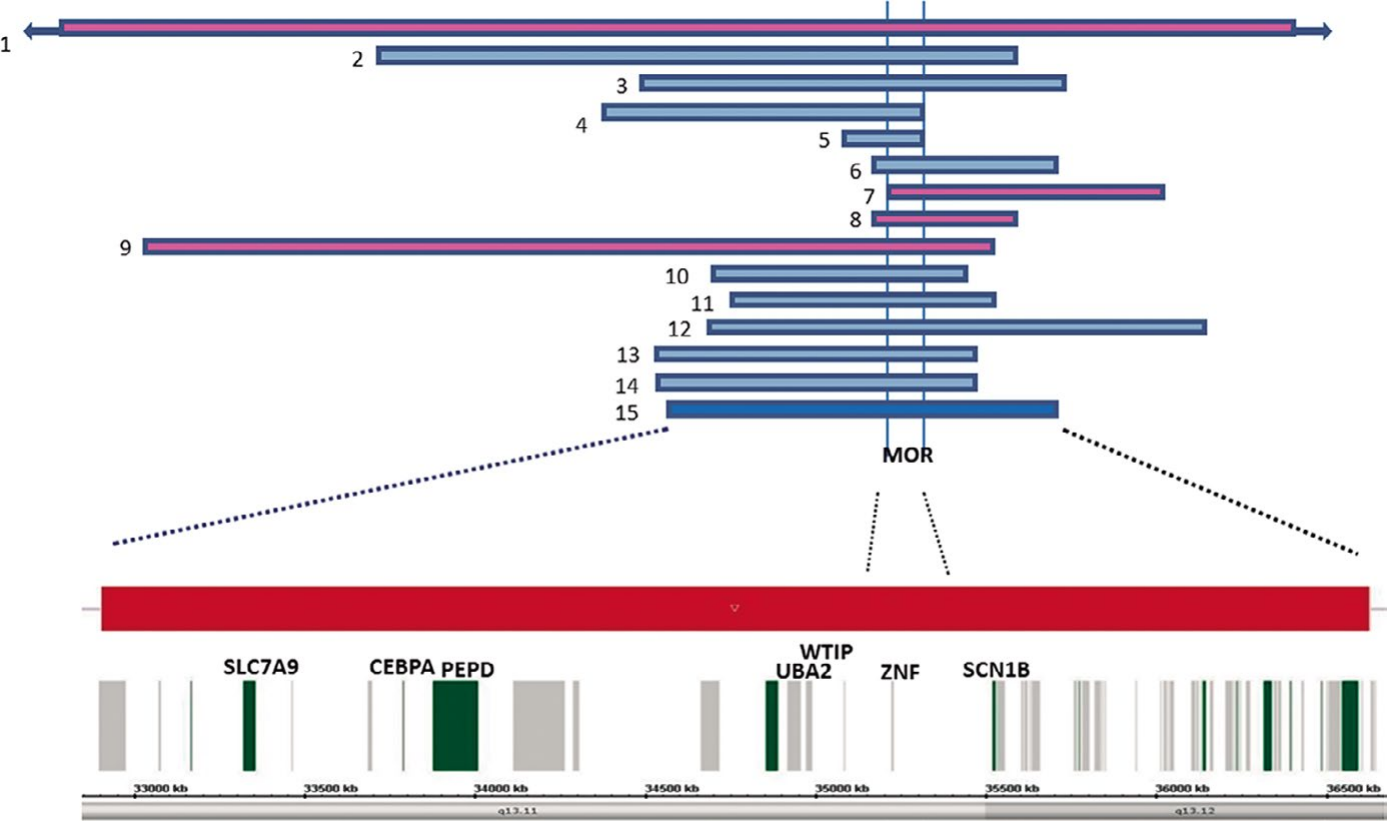
Schematic representation of the 19q13 deletion region, according to genome assembly hg19 (GRCh37). 1‐ Kulhayra et al (1998), 2‐ Malan et al (2009) (patient 1), 3‐ Malan et al (2009) (patient 2), 4‐ Malan et al (2009) (patient 3), 5‐ Schuurs‐Hoeijnakers et al (2009), 6‐ Gana et al (2012) (patient 1), 7‐ Gana et al (2012) (patient 2), 8‐ Forzano et al (2012), 9‐ Chowdhurry et al (2013) (patient 1), 10‐ Chowdhurry et al (2013) (patient 2), 11‐ Venegas‐Veja et al (2014), 12‐ Melo et al (2015), 13 and 14‐ Urquhart et al (2015), 15‐present patient. Pink line: female; blue line: male; dark blue: propositus. MOR, minimal overlapping region

Our patient shared several main features with previously reported 19q13.11 microdeletion syndrome patients, including intrauterine and postnatal growth retardation, microcephaly, developmental delay/intellectual disabilities, language delay, feeding difficulties, cutis aplasia, and hypospadias (Table 1). In addition, we confirmed the occurrence of syndactyly, brain abnormalities, and ectrodactyly less frequently. This interstitial deletion of the 19q13.11 region includes the smallest region of overlapping of the previously described patients (Figure 3).

**Table 1:** Clinical features in patients with 19q13.1 deletion

	Kulharya et al, (1998)	Malan et al, (2009)			Schuurs‐Hoeijmakers et al (2009)	Gana et al, (2012)		Forzano et al, (2012)	Chowdhury et al (2013)		Ven‐Vega et al, (2014)	Melo et al (2015)	Urquhart et al (2015)		Present report	Total
		Pat 1	Pat 2	Pat 3		Pat 1	Pat 2		Pat 1	Pat 2			Pat 1	Pat 2		
Approximated deletion size (Mb)	11	6.16	4.27	3.14	2.40	1.74	2.63	1.37	8.16	2.30	2.49	4.91	3.22	3.22	3.72	
Gender	Female	Male	Male	Male	Male	Male	Female	Female	Female	Male	Male	Female	Male	Male	Male	5 Female/10 Male
Preterm delivery (<37 wk)	+	+	+	+	+	+	+	+	nm	nm	+	−	−	−	+	76.9% (10/13)
Weight at birth (kg)	1.29	1.56	1.35	1.93	1.62	1.59	1.9	1.58	<10%	<10%	+	1.95	1.98	1.79	1.79	
Age at diagnosis (y, mo)	3 y 0 mo	6 y 0 mo	9 y 2 mo	5 y 0 mo	4 y 10 mo	14 y 0 mo	8 y 0 mo	6 y 5 mo	5 y 6 mo	1 y 6 mo	6 y 7 mo	0 y 7 mo	44 y 0 mo	44 y 0 mo	10 y 2 mo	
Developmental characteristics																
Intrautrine growth retardation	+	+	+	+	+	+	+	+	−	+	+	+	+	+	+	93.3% (14/15)
Postnatal growth retardation	+	+	+	+	+	+	+	+	+	+	+	+	+	+	+	100% (15/15)
Slender habitus	+	+	+	+	−	+	+	+	nm	nm	+	+	+	+	+	92.3% (12/13)
DD/intellectual disabilities	+	+	+	+	+	+	+	+	+	+	+	+	+	+	+	100% (15/15)
Language delay	+	+	+	+	+	+	+	+	+	+	+	+	+	+	+	100% (15/15)
Feeding difficulties	+	+	+	+	+	+	−	+	+	+	+	+	+	+	+	93.3% (14/15)
Microcephaly	+	+	+	+	+	+	+	+	+	+	+	+	+	+	+	100% (15/15)
Brain abnormalities*	−	nm	−	−	nm	+ *1	nm	+ *2	−	nm	nm	nm	nm	nm	+ *3	42.8% (3/7)
Dystonia	nm	nm	nm	nm	nm	+	−	nm	−	−	nm	+	nm	nm	−	33.3% (2/6)
Epilepsy	nm	nm	nm	nm	nm	+	−	+	−	+	+	−	−	−	−	40% (4/10)
Ectodermal dysplasia																
Hair/eyebrows/eyelashes anomalies	−	+	+	+	+	+	+	+	+	−	+	+	+	+	−	80.0% (12/15)
Thin/dry skin	−	+	+	−	+	−	−	+	−	−	+	+	+	+	−	53.3% (8/15)
Cutis aplasia in midline scalp	+	+	+	+	+	+	−	+	−	+	+	+	−	−	+	73.3% (11/15)
Dysplastic nails	−	+	+	−	+	−	nm	+	−	−	+	+	−	−	−	42.8% (6/14)
Overlapping of the toes	+	+	−	−	−	−	−	+	nm	nm	+	+	−	−	−	38.5% (5/13)
Physical abnormalities																
Hypospadias	na	+	+	+	+	+	na	na	na	+	+	na	+	+	+	100% (10/10)
Fingers/toes syndactily	−	−	+	+	+	+	−	−	+	+	+	−	−	−	+	53.3% (8/15)
Hand/foot ectrodactily	−	−	−	−	−	−	−	−	−	+	−	−	−	−	+	13.3% (2/15)
Congenital heart defects	+	nm	+	nm	−	+	−	+	−	+	−	+	+	−	−	53.8% (7/13)
Other skeletal abnormalities	+	nm	+	nm	+	−	+	+	nm	nm	+	+	nm	nm	−	77.8% (7/9)
Miscellaneous**	+ **1	+ **2	+ **3	−	+ **3	+ **4	+ **5	+ **6	+ **7	−	+ **8	+ **9	+ **10	+ **11	+ **12	86.6% (13/15)
Minor dysmorphic features																
High forehead	+	+	+	+	+	+	nm	+	−	nm	+	+	+	+	+	92.3% (12/13)
Micrognatia/retrognatia	+	+	+	−	+	+	+	+	−	+	+	+	+	+	+	93.3% (14/15)
Low set ears/poorly folded ears	+	−	+	+	+	+	+	+	+	+	+	+	+	+	+	93.3% (14/15)
Thin lips	+	+	+	+	+	+	+	+	+	nm	+	+	+	+	−	92.8% (13/14)

(+) Feature present; (−) feature absent; nm: not mentioned; na: not applicable; pat: patient; brain abnormalities *: *1 mild enlargement of the lateral ventricles; *2 hypoplastic pituitary gland and thin corpus callosum; *3 encephalocele and Dandy‐Walker variation; miscellaneous **: **1 deafness and hidronephrosis; **2 single median incisor and bifid scrotum; **3 cataract; **4 growth hormone deficiency; **5 hypodontia and teeth abnormalities; **6 hypothyroidism, ACTH and GH deficiency; **7 bifid uvula; **8 bifid scrotum; **9 teeth abnormalities; **10 and **11 Strabismus, teeth abnormalities and bifid scrotum; **12 teeth abnormalities and gallblader stone; DD: Developmental delay; Ven‐Vega: Venegas‐Vega et al (2014). Clinical features of the patients related from: Kulhayra et al (1998); Malan et al (2009) (pat 1, pat 2, pat 3); Schuurs‐Hoeijmakers et al (2009); Gana et al (2012) (pat 1, pat 2); Forzano et al (2012); Chowdhury et al (2013) (pat 1, pat 2); Venegas‐Vega et al (2014); Melo et al (2015); patients 13, 14 ‐ Urquhart et al (2015) (pat 1, pat 2); current patient.

The critical and minimal overlapping region (MOR) of 324 kb encompasses four zinc finger (*ZNF302, ZNF181, ZNF599,* and *ZNF30*) genes belonging to the Kruppel‐associated box (KRAB)‐containing *ZNF* genes. Zinc finger proteins, which were widely expressed, have been shown to interact with nucleic acids and to have diverse functions, such as stimulation of transcription, transcriptional repression, binding to single‐stranded DNA, binding to RNA, or bi‐functional DNA‐ and RNA‐binding activities. Their role in the development of cognitive functions has been speculated, and their haploinsufficiency could be involved in the pathogenesis of the syndrome, as the *ZNF* cluster at X chromosome contribute to the X‐linked intellectual disabilities.^3^, ^9^, ^11^

A few genes proximal to MOR, namely, the *CEBPA* (116897), *PEPD* (613230), *WTIP* (614790) and *UBA2* (613295) genes, have been proposed to contribute to the clinical characteristics frequently observed in patients either because they are also deleted or because their regulation regions may be disrupted.^3^, ^7^ Although poorly understood, it is suggested that *CEBPA* has multiple functions in adipogenesis^12^ and shares significant overlap in its regulation and target genes with *TP63*, the heterozygous mutation of which causes EEC.^13^ *PEPD* insufficiency causes prolidase deficiency, an autosomal recessive disorder with a variety of signs, including distinctive face, mental retardation, and chronic recurrent cutaneous ulcers.^14^ The *CEBPA* or *PEPD* deletion was presented nearby 70% of the reviewed patients with ectodermal dysplasia (Table S1). The most consistent finding was the observation of hypospadias and the deletion of *UBA2* and *WTIP* genes in all male individuals with this syndrome.^2^, ^3^, ^5^, ^6^, ^8^, ^9^ UBA2 protein forms a heterodimer that behaves as a SUMO‐activating (small ubiquitin‐like modifier‐activating) enzyme for the post‐translational modification of proteins. Several transcription regulators, hormone receptors, and signaling proteins are regulated and affected by this post‐translational modification. Thus, it has been suggested that SUMO modification may represent a common pathway that regulates normal craniofacial, genital development and is involved in the pathogenesis of cutis aplasia, orofacial clefting, and hypospadias.^5^, ^7^, ^9^, ^15^ Corroborating this theory, the *UBA2* deletion was observed in 76% of the patients with cutis aplasia. *WTIP* is expressed in a variety of tissues and has an important role in renal development and the maintenance of glomerular podocytes. In addition, its association with hypospadias has been proposed.^5^, ^6^ Some other genes proximal to MOR have been associated with less common clinical manifestations in a few cases, depending on the region deleted or controlling factors: cardiac conduction disturbance or generalized epilepsy related to mutations in the *SCN1B* gene; dystonia caused by haploinsufficiency of *KMT2B* gene; renal anomalies associated with the *USF2* gene.^7^, ^16^, ^17^, ^18^, ^19^, ^20^ Between fifteen reviewed patients, 54% of them with the *SCN1B* deletion presented congenital heart defects and 31% epilepsy, 25% with the *KMT2B* deletion were dystonic and just 8% with the *USF2* deletion had hydronephrosis.

The identification of both symptomatic and asymptomatic carriers suggests the possibility that other genetic, epigenetic or environmental modifiers influence disease penetrance and phenotypic presentation. Imprinted genes, parental mosaicism, regulator genes, and true incomplete disease penetrance have been considered in research.^18^, ^21^ Our survey reinforces that more careful clinical and molecular characterization are needed to delineate the phenotype‐genotype correlation and the minimal critical region of 19q13.11 microdeletion syndrome.

Cytogenetic molecular techniques, such as comparative genomic hybridization (CGH) and chromosome microarray (CMA), have elucidated some disorders that were previously unclassified or misclassified due to clinical overlapping.^8^ Here, in our report, we described a child whose diagnosis was EEC syndrome before receiving microarray results. EEC syndrome is a genetically heterogeneous disorder with marked phenotypic variability. The major features comprise absence of the central parts of the hands and feet, resulting in split hand/foot malformation (84%), ectodermal dysplasia (77%), and cleft lip with or without cleft palate (68%).^22^ Individuals with EEC syndrome can also develop a variety of additional symptoms, including abnormalities of the genitourinary system and the eyes. The majority of cases appear to be secondary to mutation in the *TP63* gene, located at 3q37. In rare cases, individuals with EEC syndrome carry chromosomal disruptions (deletions, translocations) on the long arm of chromosome 7 (7q11.2‐q21.3). O'Quinn et al^23^, 1998, described a family with EEC syndrome and proposed a new candidate gene *TGF‐ß1*, located at 19q13.1. Recently, Kosaki et al^24^, 2012 reported on a patient with EEC‐like features and an *IRF6* (region at 1q32.2) mutation and suggested that a protein–protein interaction occurs between *IRF6* and *TP63*. We noticed considerable overlap between 19q13.11 microdeletion syndrome and EEC syndrome, and we speculated that haploinsufficiency of genes such as *CEBPA* and *PEPD,* which are proximal to MOR, may contribute to EEC phenotype. However, additional studies are necessary to confirm the association between the deletion of these genes and clinical manifestations.

In conclusion, we reported an additional patient affected by 19q13 microdeletion syndrome whose characteristics may contribute to a review of the minimal associated region and may focus on a few genes that are probably responsible for the phenotype. In addition, we suggest that suspected cases of EEC syndrome could be tested for 19q13.11 microdeletion to search for genotype‐phenotype correspondence.

## CONFLICT OF INTEREST

None declared.

## AUTHORSHIP

KTA and IMPOR: acquired the data, analyzed and interpreted the data, drafted and finalized the manuscript. NSJr: ran the testing and contributed to the molecular portion of the study and critically revised the manuscript. ALVC, DRC, and CES‐M: involved in the clinical management of the patient, performed all the clinical investigations and revised the manuscript.
