# Supplementary material for: 19q13.11 microdeletion: Clinical features overlapping ectrodactyly ectodermal dysplasia‐clefting syndrome phenotype
Source: Clin Case Rep. 2018 May 28;6(7):1300–7. doi: 10.1002/ccr3.1600 (PMC6028370; doi:10.1002/ccr3.1600)
Supplement: Supplementary file 1 [file CCR3-6-1300-s001.docx]

| Authors | | |  | | |  | | | Kulharya et al., 1998 | | Malan et al., 2009 | |  |  | S-H et al., 2009 | Gana et al., 2012 |  |
| --- | --- | --- | --- | --- | --- | --- | --- | --- | --- | --- | --- | --- | --- | --- | --- | --- | --- |
|  | | |  | | |  | | |  |  | patient 1 | | patient 2 | patient 3 |  | patient 1 | patient 2 |
|  | | |  | | | **Genomic position hg19** | | |  | | 30,102,849-36,262,996 | | 32,609,986-36,885,340 | 32,293,672-35,440,134 | 34,686,784-35,440,134 | 34,934,735-36,639,035 | 35,111,811-37,744,992 |
| **Patients** | | |  | | |  | | | 1 | | 2 | | 3 | 4 | 5 | 6 | 7 |
| **Deletion size (Mb)** | | |  | | |  | | | 11 | | 6,16 | | 4,27 | 3,19 | 2,4 | 1,74 | 2,63 |
| **Gender** | | |  | | |  | | | Female | | Male | | Male | Male | Male | Male | Female |
| **Associations according to the literature** | | |  | | |  | | |  | |  | |  |  |  |  |  |
| Gene | | | *CEBPA* (OMIM 116897) | | | 33,790,839-33,793,469 | | | deleted | | deleted | | deleted | deleted | not deleted | not deleted | not deleted |
| Phenotype | | | cutis aplasia in midline scalp | | |  | | | + | | + | | + | + | + | + | - |
| Gene | | | *PEPD* (OMIM 613230) | | | 33,877,856-34,012,700 | | | deleted | | deleted | | deleted | deleted | not deleted | not deleted | not deleted |
| Phenotype | | | cutis aplasia in midline scalp | | |  | | | + | | + | | + | + | + | + | - |
| Gene | | | *WTIP* (OMIM 614790) | | | 34,972,839-35,000,051 | | | deleted | | deleted | | deleted | deleted | deleted | deleted | not deleted |
| Phenotype | | | hypospadia | | |  | | | na | | + | | + | + | + | + | na |
| Gene | | | *UBA2* (OMIM 613295) | | | 34,919,080-34,960,797 | | | deleted | | deleted | | deleted | deleted | deleted | deleted | not deleted |
| Phenotype | | | hypospadia | | |  | | | na | | + | | + | + | + | + | na |
| Gene | | | *UBA2* (OMIM 613295) | | | 34,919,080-34,960,797 | | | deleted | | deleted | | deleted | deleted | deleted | deleted | not deleted |
| Phenotype | | | cutis aplasia in midline scalp | | |  | | | + | | + | | + | + | + | + | - |
| Gene | | | *SCN1B* (OMIM 600235) | | | 35,521,591-35,531,352 | | | deleted | | deleted | | deleted | not deleted | not deleted | deleted | deleted |
| Phenotype | | | congenital heart disease | | |  | | | + | | nm | | + | nm | - | + | - |
| Phenotype | | | epilepsy | | |  | | | nm | | nm | | nm | nm | nm | + | - |
| Gene | | | *KMT2B* (OMIM 606834) | | | 36,208,719-36,229,780 | | | deleted | | deleted | | deleted | not deleted | not deleted | deleted | deleted |
| Phenotype | | | dystonia | | |  | | | nm | | nm | | nm | nm | nm | + | - |
| Gene | | | *USF2* (OMIM 600390) | | | 35,759,880-35,770,723 | | | deleted | | deleted | | deleted | not deleted | not deleted | deleted | deleted |
| Phenotype | | | hypospadia | | |  | | | na | | + | | + | + | + | + | na |
| Phenotype | | | hydronephrosis | | |  | | | + | | nm | | nm | nm | nm | nm | nm |
| Forzano et al., 2012 | Chowdhury et al 2013 |  | | Venegas-Vega et al., 2014 | Melo et al., 2015 | | Uruquhart et al., 2015 | | | Present | |  |  |  |  |  |  |
|  | patient 1 | patient 2 | |  |  |  | patient 1 | patient 2 | | patient | |  |  |  |  |  |  |
| 34,983,674-36,361,210 | 27,853,206-36,012,094 | 33,421,401-35,726,387 | | 33,565,628-36,055,467 | 33,203,635-38,108,990 | | 32,773,586-35,998,214 | 32,773,586-35,998,214 | | 32,904,200-36,627,790 | |  |  |  |  |  |  |
| 8 | 9 | 10 | | 11 | 12 | | 13 | 14 | | 15 | |  |  |  |  |  |  |
| 1,37 | 8,16 | 2,3 | | 2,49 | 4,9 | | 3,2 | 3,2 | | 3,7 | |  |  |  |  |  |  |
| Female | Female | Male | | Male | Female | | Male | Male | | Male | |  |  |  |  |  |  |
|  |  |  | |  |  | |  |  | |  | |  |  |  |  |  |  |
| not deleted | deleted | deleted | | deleted | deleted | | deleted | deleted | | deleted | |  |  |  |  |  |  |
| + | - | + | | + | + | | - | - | | + | |  |  |  |  |  |  |
| not deleted | deleted | deleted | | deleted | deleted | | deleted | deleted | | deleted | |  |  |  |  |  |  |
| + | - | + | | + | + | | - | - | | + | |  |  |  |  |  |  |
| deleted | deleted | deleted | | deleted | deleted | | deleted | deleted | | deleted | |  |  |  |  |  |  |
| na | na | + | | + | na | | + | + | | + | |  |  |  |  |  |  |
| not deleted | deleted | deleted | | deleted | deleted | | deleted | deleted | | deleted | |  |  |  |  |  |  |
| na | na | + | | + | na | | + | + | | + | |  |  |  |  |  |  |
| not deleted | deleted | deleted | | deleted | deleted | | deleted | deleted | | deleted | |  |  |  |  |  |  |
| + | - | + | | + | + | | - | - | | + | |  |  |  |  |  |  |
| deleted | deleted | deleted | | deleted | deleted | | deleted | deleted | | deleted | |  |  |  |  |  |  |
| + | - | + | | - | + | | + | - | | - | |  |  |  |  |  |  |
| + | - | + | | + | - | | - | - | | - | |  |  |  |  |  |  |
| deleted | not deleted | not deleted | | not deleted | deleted | | not deleted | not deleted | | deleted | |  |  |  |  |  |  |
| nm | - | - | | nm | + | | nm | nm | | - | |  |  |  |  |  |  |
| deleted | deleted | not deleted | | deleted | deleted | | deleted | deleted | | deleted | |  |  |  |  |  |  |
| na | na | + | | + | na | | + | + | | + | |  |  |  |  |  |  |
| nm | nm | nm | | nm | nm | | nm | nm | | - | |  |  |  |  |  |  |

Supplementary table: clinical features in patients with deletion 19q13.11 and genotypes (part 1 and 2). (+) feature presente; (-) feature absent; nm: not mentioned; na: not applicable
